# Frequency of secondary modifiers in Beta Thalassemia intermedia in patients from Northern Punjab

**DOI:** 10.12669/pjms.39.5.7376

**Published:** 2023

**Authors:** Fariha Nasreen, Attika Khalid, Lubna Zafar, Suhaib Ahmad, Asma Shaikh

**Affiliations:** 1 Dr. Fariha Nasreen, FCPS (Heam) Wapda Hospital, Rawalpindi, Pakistan; 2 Dr. Attika Khalid, FCPS (Haem) Pathology Department, Foundation University Medical College and Hospital, Islamabad, Pakistan; 3 Prof. Lubna Zafar, FCPS (Haem) Pathology Department, Foundation University Medical College and Hospital, Islamabad, Pakistan; 4 Prof. Suhaib Ahmed, FCPS (Haem), PhD. Riphah International University, Islamabad, Pakistan; 5 Dr. Asma Shaikh, MPhil (Haem) Sindh Medical Colleague, Jinnah Sindh Medical University, Karachi, Pakistan

**Keywords:** Xmn-l, Polymorphism, Thalassemia intermedia

## Abstract

**Objective:**

The aim of this study was to determine frequency of secondary modifiers in non-transfusion dependent thalassemia.

**Methods:**

This descriptive cross sectional study was done at Fauji Foundation Hospital Islamabad. Seventy diagnosed patients of thalassemia intermedia were included. Deoxyribonucleic acid (DNA) was extracted using Chelex method. The Xmn-1 and BCL11A polymorphisms were analyzed by Amplification Refractory Mutation System (ARMS) and Restriction Fragment Length Polymorphism (RFLP) PCR. The PCR amplified products were run on Polyacrylamide Gel Electrophoresis (PAGE).

**Results:**

The Xmn-l polymorphism was seen in 26/70 (37.1%) and BCL11A polymorphism was seem in 50/70 (71.4%) of the patients.

**Conclusion:**

BCL11A and Xmn-l polymorphisms are important secondary modifiers in patients with thalassaemia intermedia in Northern Punjab.

## INTRODUCTION

Thalassemiaare a group of inherited disorders that are either due to a defect in the structure of hemoglobin or its synthesis.^1^,^2^ These are the most common monogenic disorders worldwide thus account for significant mortality and morbidity all over the world.^1^,^2^

Clinically Thalassemia can be categorized as thalassemia major, intermedia and trait clinically.^1^,^2^ Thalassemia major is the grave condition usually presented by progressive anemia, failure to thrive and lifelong dependency on frequent blood transfusions.^1^ Regular blood transfusions result in iron overload and other metabolic complications that further increases mortality and morbidity. Bone marrow transplantation is the sole therapeutic treatment but it is not easily available in Pakistan and majority of patients cannot afford it.^3^ Thalassemia trait, the mildest form, is a carrier state and remains asymptomatic without requiring any treatment most of the time.^1^

Thalassemia intermedia also known as non-transfusion dependent thalassemia (NTDT) is a clinical condition that vary in severity from symptomatic carriers to transfusion dependent patients.^4^,^5^ Estimated prevalence of thalassemia intermedia is approximately 5.73% in Pakistan.^4^ Patients with thalassemia intermedia are distinguish by varying degree anemia, mild jaundice and usually do not have bony deformities or thalassemic facies. They do not need blood transfusion routinely but only in stressful conditions. They have overall greater survival time and relatively better quality of life.^1^,^2^,^4^ The clinical difference from thalassemia major makes it necessary to distinguish between the two forms to allow for appropriate treatment and timely control of complications due to unnecessary transfusions.^4^

A number of genetic factors affect the hematological parameters and clinical course of NTDT. Various genetic modifiers at different levels affect the phenotypic diversity of thalassemia intermedia. These modifiers can be primary, secondary and tertiary.^1^ Primary modifiers are the genetic mutations that affects beta globin chain synthesis. These mutations lead to variable expression of globin genes leading to absent or a very low level of globin chain. Secondary modifiers are those factors that reduce the degree of imbalance of globin chain synthesis. These include co-inheritances of alpha thalassemia, increased production of gamma chains, mutation associated with increase production of HbF like Xmn l and BCL11A polymorphism.^2^,^5^ Tertiary modifiers are the factors that are not directly involved in globin chain synthesis. They effect disease complications by affecting the genes which are related with absorption of iron, metabolism of bilirubin and bone, cardiovascular disease and venerable to infections.^6^

More than 200 point mutations are reported as a cause of beta thalassemia. However, genotypic variability is inadequate to clarify the phenotypic variability among individuals having identical genotype.^3^ Therefore, researchers are more towards on identifying genetic modifiers for severity in thalassemia in order to design more distinct and useful therapies. The aim of this study was to find out frequency of secondary modifiers in NTDT so that more specific and effective management can prevent various complications in our region from unnecessary transfusions.

## METHODS

Descriptive cross sectional study was done at Fauji Foundation Hospital Islamabad. A total of 70 diagnosed patients of thalassemia intermedia were enrolled in this research. Ethical approval was taken by ethical review committee of Fauji Foundation Hospital. Study was carried out from June 2021 to January 2022.

### Inclusion criteria:

Diagnosed patients of Beta thalassemia intermedia. All ages and both genders were included.

### Exclusion Criteria:

All forms of thalassemia other than beta thalassemia intermedia were excluded.

After taking informed consent and detail history of all patients, 5ml venous blood was taken in EDTA (Ethylene Diamine Tetra Acetic Acid) container. Deoxyribonucleic acid (DNA) was obtained by utilizing Chelex method. PCR was carried out.

### BCL11A polymorphism:

Single nucleotide polymorphism of rs11886868 in the BCL11A gene (T→C) was determined by PCR-RFLP technique. A 548bp fragment was amplified using the primers 5’-TTTGGTGCTACCCTGAAAGAC-3’ and 5’-ACTCAACAGTAGCAGAATGAAAGAG-3

The 548-bp product was digested with MboII restriction enzyme by incubating at 37°C for five minutes, followed by separation of fragments on 6% polyacrylamide gels. The T allele lacks the MboII restriction site, while it is present in the C allele. The C allele was detected as two fragments of 470 bp and 70 bp.

### Xmn-1 Polymorphism:

Xmn-1 polymorphism was done by using Amplification Refractory Mutation System (ARMS) method. The following primers were used:

Xmn-I-Common 5’-CCCATGGCGTCTGGACTAG

Xmn-I-Normal 5’-TGCAAATATCTGTCTGAAACGATC

Xmn-I-Mutant 5’-TGCAAATATCTGTCTGAAACGATT

The PCR for ARMS was done by using a 25μl reaction mixture containing 5 pM of each primer, 0.5 units of Taq polymerase (Thermo Fisher Scientific, USA), 30 µM of each dNTP (Thermo Fisher Scientific, USA), 10 mM Tris HCl (pH 8.3), 50 mM KCL, 1.5 mM MgCl_2_, 100 mg/ml gelatin (Sigma, UK), and 0.3-0.5 µg of genomic DNA. Primers were manufacture by Macrogen, (Korea). Automated DNA thermal cycler Gene Amp 9700 (ABI, USA) was used for thermal cycling. The regimen consisted of 25 cycles each consisting of denaturation at 94^o^C for one minute, primer annealing at 65^o^C for one minute, and DNA extension at 72^o^C for 1½ minutes. In the final cycle, the extension reaction was prolonged for another three minutes. The amplified product was run on Mini Poly Acrylamide Gels (PAGE).

### Statistical analysis:

Data analysis was done by applying SPSS version 17. Frequency and percentages were assessed for qualitative variables like gender of patient and secondary modifiers. Mean and standard deviation were determined for quantitative variables like age of patient.

## RESULTS

A total of 70 patients of beta thalassemia intermedia were studied, 41 (58.57%) were female and 29 (41.43%) were males with proportion of 1:1.4. In this research, age varying between 3-30 years and mean age was 14.81 ± 6.15 years. The frequency of secondary modifiers in beta-thalassemia intermedia were found to be: Xmn l polymorphism in 26/70 (37.14%) patients and BCL 11A gene in 50/70 (71.43%) patients as shown in Table-I.

**Table-I:** Frequency of secondary modifiers in beta-thalassemia intermedia

Secondary Modifiers	Frequency (%)	

	Detected	Not detected
Xmn l polymorphism	26/70 (37.14%)	44/70 (62.86%)
BCL 11A gene	50/70 (71.43%)	20/70 (28.57%)
Xmn 1 +BCL 11A (both detected)	18/70 (26%)	-
Xmn 1 +BCL 11A (both not detected)	-	10/70(14.2%)

## DISCUSSION

The clinical course of the disease in homozygous beta-thalassemia is affected by the presence of secondary genetics modifiers. Modifier genes are genetic variants that affect the phenotypic presentation of any disorder.^7^ Secondary modifiers are a set of genes that are linked with elevated levels of HbF or increase production of γ chain therefore improving α / non α globin chain imbalance. These include Xmn l, BCL11A, HBS1LMYB and other genes. The globin chain imbalance which is a major factor in pathogenesis of thalassemia major, can be reduced by these genetic variants. This results in clinically milder form of thalassemia that is thalassemia intermedia.^8^,^9^ Current study reports the frequency of Xmn I and BCL11A in Northern Punjab in Pakistan. The reason for selecting these two genes are availability of primers and technical expertise. Moreover, there is scanty data available on the incidence of BCL11A and Xmn-I in thalassemia intermedia in Pakistan.

Age range in this study was 3-30 years with mean age of 14.81 ± 6.15 years. Majority of the patients 37 (52.86%) were between 3-15 years of age. Out of the 70 patients with thalassemia intermedia, 29 (41.43%) were male and 41 (58.57%) were females and male to female proportion of 1:1.4.

Our study showed 37.14% frequency of Xmn I in Northern Punjab in Pakistan. However, in a local study conducted in northern Pakistan the prevalence of Xmnl polymorphism in homozygous/compound heterozygous beta thalassemia group was found to be 13%.^10^ Study conducted in India observed XmnI polymorphism homozygosity in 27.4% of patients.^8^ These results are comparable to our study. The reason for the discrepancy between the frequency of Xmnl polymorphism can be the genetic distribution of polymorphism and sample size not linked to the type of beta thalassemia mutations.

The prevalence of heterozygote XmnI gene was 11.6% in thalassemia intermedia patients in a study conducted in Egypt which is notably lower than current study.^11^ The prevalence of Xmn1 gene polymorphism in a study conducted in north of Iran was 86%, which is either in one locus (- / +, 21%) or both loci (+/+, 65%).^12^ An other study from Iran by Miri-Moghaddam et al. showed frequency of Xmn1 Gene Polymorphism in South East of Iran to be 62%.^13^ This could be owing to the fact that the Xmn1 polymorphism has a diverse geographical distribution across patients from different nations as well as different regions within same countries.

The frequency of BCL11A in our study was 71.43%. There is scanty data available for frequency of BCL11A polymorphism in Pakistan. A study conducted in Khyber Pakhtunkhwa Pakistan detected BCL11A in 70.3% of thalassemic patients.^14^ Comparable result was also shown in a study done on Sardinian population.^15^ It was also discovered that mutations at the BCL11A gene affect HbF levels, positively influencing the clinical presentation of thalassemia. Most global studies have recorded variant genotype of BCL11A rather than BCL11A mutation as a whole.^16^,^17^

In India prevalence of CC and TC variant genotype of BCL11A was 51.2% and 40.0% respectively.^16^ In another study the incidence of the CC genotype was shown to be 28.8% in an Iranian population.^17^ According to Galanello *et al* CC genotype is present in 61.2 percent of Sardinians.^18^ The variation in the frequency of variant genotype could be due to some ethnic variations. We studied the overall frequency of BCL11A not its variant genotype. The frequency of BCL11A is on higher side in thalassemia patients.

### Limitations of study:

This study is a single region study and sample size is small.

## CONCLUSION

BCL11A and Xmn-l polymorphisms are important secondary modifiers in patients with thalassaemia intermedia in Northern Punjab. These markers can be used for early diagnosis and proper management of thalassemia intermedia patients.

### Authors’ Contributions:

**FN:** Sample collection, Writing. Literature search, Data analysis and Interpretation.

**AK:** Interpretation of Data, Questionnaire design, Revising of article.

**LZ:** Study Design and concept. Final review. Supervision of all research work, Interpretation of Data.

**SA:** Final review, supervision.

**AS:** Writing, Literature search, data analysis, drafting.
